# Uterine hypertonus and fetal bradycardia occurred after combined spinal-epidural analgesia during induction of labor with oxytocin infusion: A case report

**DOI:** 10.1097/MD.0000000000016282

**Published:** 2019-07-12

**Authors:** Lingyun Yang, Li Wan, Han Huang, Xiaorong Qi

**Affiliations:** a Department of Gynecology and Obstetrics, West China Second University Hospital, Sichuan University; b Key Laboratory of Birth Defects and Related Diseases of Women and Children, Sichuan University, Ministry of Education; c Department of Anaesthesiology, West China Second University Hospital, Sichuan University, Chengdu, China.

**Keywords:** combined spinal-epidural analgesia, fetal bradycardia, oxytocin infusion, uterine hypertonus

## Abstract

**Rationale:**

Pain management is an essential part of good obstetrical care. The rapid onset of pain relief after combined spinal-epidural (CSE) analgesia may cause a transient imbalance in maternal catecholamine level, leading to uterine hyperactivity and fetal heart rate (FHR) abnormalities. How to manage the uterine basal tone and FHR abnormalities after labor analgesia still remains controversial.

**Patient concerns:**

A 33-year-old nulliparous woman at 40^+5^ weeks’ gestation underwent induction of labor after premature rupture of membranes. CSE analgesia was provided when the patient described her pain as the top on a scale of 10 during induction of labor with oxytocin infusion.

**Diagnoses:**

Uterine hypertonus and fetal bradycardia were diagnosed within 10 minutes after CSE analgesia.

**Interventions:**

Oxytocin infusion and CSE analgesia were immediately suspended, and measures of staying in left lateral decubitus position and giving supplemental oxygen were attempted to resuscitating the baby. Because of suspicious fetal distress, the baby was rapidly delivered by emergency cesarean section.

**Outcomes:**

The Apgar score of the baby was 8 and 10 at 1 and 5 minutes after birth. Subsequent follow-up confirmed that both mother and baby were in good condition.

**Lessons:**

The loss of the tocolytic effect of epinephrine after CSE analgesia and continuous oxytocin infusion may work together to form a totally synergistic function, finally leading to inevitable uterine hypertonus and fetal bradycardia. Both the obstetrical provider and anesthesiologist should carefully monitor all patients in the first 15 minutes after CES analgesia induction. Oxytocin administration in this critical period deserves attention. Additionally, intraprofessional collaboration is also necessary to ensure high quality and safe delivery for all childbearing women.

## 1 Introduction

For most women labor causes severe pain caused by uterine contractions and cervical dilatation. Neuraxial labor analgesia has been shown to be effective for alleviating labor pain.^[1]^ However, it is reported that the rapid onset of analgesia may cause transient imbalance in maternal catecholamine level, leading to uterine hyperactivity and fetal heart rate (FHR) abnormalities, which is a concern for patients who desire to have pain relief during childbirth. How to manage the uterine basal tone and FHR abnormalities after labor analgesia still remains controversial.^[2]^ We herein present a case that uterine hypertonus and fetal bradycardia occurred after combined spinal-epidural (CSE) analgesia during induction of labor with oxytocin infusion. We considered that the loss of the tocolytic effect of epinephrine after CSE analgesia and continuous oxytocin infusion worked together to form a totally synergistic function, finally leading to inevitable uterine hypertonus and fetal bradycardia. Consequently, this case is being reported to suggest that obstetrical provider and anesthesiologist should carefully monitor all patients in the first 15 minutes after CES analgesia induction. Oxytocin administration in this critical period deserves attention.

## 2 Case report

Ethical approval and patient consent were acquired and recorded in the patient medical record with witness signature. All ethical approval and consent procedures were approved by the Medical Ethical Committee of West China Second University Hospital, Sichuan University (Ethical approval No. 2018059).

A 33-year-old nulliparous woman at 40^+5^ weeks’ gestation presented to our hospital and underwent induction of labor because of premature rupture of membranes. Her pregnancy course was uncomplicated and general examination findings were normal. She received an oxytocin infusion with the dose increasing from 12 ml per hour to 72 ml per hour. As labor progressed after 8 hours induction, her cervical dilatation was 3 cm and the descending of the fetal head went well with the position at −1 station. Meanwhile, the patient described her pain as the top on a scale of 10, and she strongly preferred a vaginal delivery. Consequently, the anesthesiologist was consulted to conduct CSE analgesia during labor and delivery.

**Figure 1:**
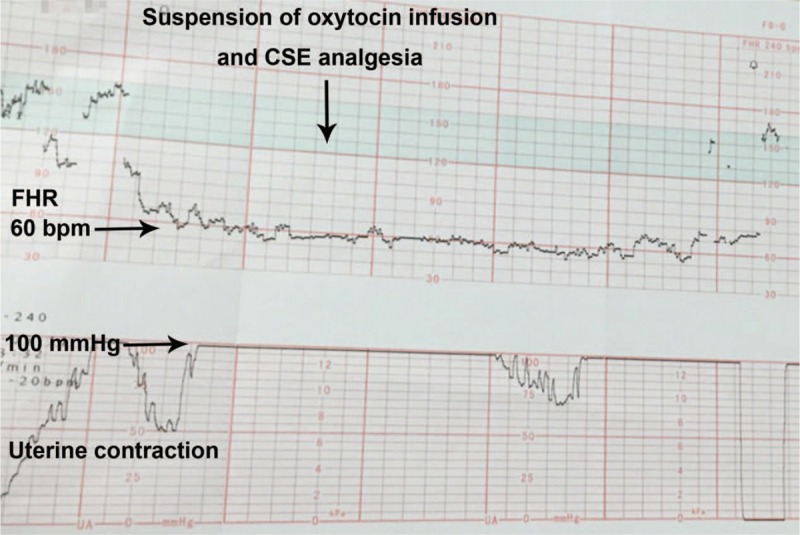
Tracing of FHR and uterine contraction on the electronic fetal monitor after CSE analgesia. Uterine hypertonus and severe fetal bradycardia could not alleviated after suspending oxytocin infusion and CSE analgesia. CSE = combined spinal-epidural, FHR = fetal heart rate.

The patient received an intrathecal injection of 0.5% bupivacaine 2.5 mg plus fentanyl 15 mg, followed by placement of an epidural catheter with the needle-through-needle technique. The labor pain was rapidly relieved in 5 minutes. Within 10 minutes of CSE analgesia, uterine hypertonus and severe fetal bradycardia (FHR <70 beats per minute) were observed on the electronic fetal monitor (Fig. 1). Oxytocin infusion and CSE analgesia were immediately suspended. Meanwhile, the patient was placed in the left lateral decubitus position and given supplemental oxygen without improvement of FHR and alleviation of uterine hyperactivity. Because of the non-reassuring FHR and suspicious fetal distress, the patient was transferred to the operating room immediately. In consultation with the obstetrical team, it was decided that an emergency cesarean delivery with general anesthesia would be performed. The baby was rapidly delivered within 2 minutes after induction of anesthesia and amniotic fluid at the time of uterine incision was frankly meconium-stained. The Apgar score was 8 and 10 at 1 and 5 minutes after birth and the baby weighed 2790 g. The patient was discharged home on her 3 postpartum days without any complications. Subsequent follow-up confirmed that both mother and baby were in good condition.

## 3 Discussion

Pain management is an essential part of good obstetrical care. In our practice, CSE analgesia is frequently provided when rapid pain relief is desired or when the request is made in advanced labor. The increasingly popular CSE technique with addition of opioids was associated with elevation of uterine basal tone and FHR abnormalities.^[3]^ It is reported that 12% FHR abnormalities with only 2% uterine hyperactivity in CSE group receiving bupivacaine with sufentanil.^[4]^ Another study demonstrated that 28% FHR changes and 8% simultaneous uterine tone elevation observed and recorded after receiving intrathecal 5 mg sufentanil and 1 mg bupivacaine.^[5]^ Effective and rapid pain relief using intrathecal fentanyl significantly decreased circulating catecholamine level, especially epinephrine, but not norepinephrine, which may be responsible for increased uterine tone.^[6,7]^ That is why the rapid onset of analgesia could be associated with uterine hyperactivity. It is noteworthy that CSE analgesia does increase the risk of emergency cesarean delivery for fetal bradycardia compared to intravenous meperidine.^[8]^ In this case, uterine hypertonus could not alleviated spontaneously after suspending oxytocin infusion and CSE analgesia. We considered that the loss of the tocolytic effect of epinephrine after CSE analgesia and continuous oxytocin infusion worked together to form a totally synergistic function, finally leading to inevitable uterine hypertonus and fetal bradycardia. Besides, more investigations are needed to better understand the effects of regional analgesia and oxytocin on labor progress and fetal physiology.

Generally, induction of labor with oxytocin infusion is monitored by obstetrical providers (including obstetricians and midwives), who may ignore the loss of the tocolytic effect of catecholamine after CSE analgesia. While labor analgesia is provided by anesthesiologists, who lack experiences in labor management. The vacancy of intraprofessional collaboration and systems optimization, in certain situations, may negatively affect the course of labor and neonatal outcome. It is reports that women presented uterine hypertonia and fetal bradycardia during the first 15 minutes after CSE analgesia.^[2]^ We suggest that both obstetrical provider and anesthesiologist should carefully monitor all patients in the first 15 minutes after CES analgesia induction. Oxytocin administration in this critical period deserves attention. In addition, anesthesiologists should look beyond their specialty and embrace the role of peridelivery physicians.^[9,10]^ Intraprofessional collaboration is also necessary to ensure high quality and safe delivery for all childbearing women.

## Author contributions

**Conceptualization:** Xiaorong Qi.

**Formal analysis:** Lingyun Yang, Li Wan.

**Investigation:** Lingyun Yang, Li Wan, Han Huang, Xiaorong Qi.

**Methodology:** Lingyun Yang, Han Huang.

**Project administration:** Xiaorong Qi.

**Writing – original draft:** Lingyun Yang, Li Wan.

**Writing – review and editing:** Xiaorong Qi.
